# Rapid, Sensitive, and Accurate Point-of-Care Detection of Lethal Amatoxins in Urine

**DOI:** 10.3390/toxins12020123

**Published:** 2020-02-15

**Authors:** Candace S. Bever, Kenneth D. Swanson, Elizabeth I. Hamelin, Michael Filigenzi, Robert H. Poppenga, Jennifer Kaae, Luisa W. Cheng, Larry H. Stanker

**Affiliations:** 1 Foodborne Toxin Detection and Prevention Research Unit, Western Regional Research Center, Agricultural Research Service, United States Department of Agriculture, 800 Buchanan Street, Albany, CA 94710, USA; candace.bever@usda.gov (C.S.B.); lstanker@gmail.com (L.H.S.); 2 Division of Laboratory Sciences, National Center for Environmental Health, Centers for Disease Control and Prevention, Atlanta, GA 30333, USA; ost3@cdc.gov (K.D.S.); ehamelin@cdc.gov (E.I.H.); 3 California Animal Health and Food Safety Laboratory System, University of California, 620 West Health Sciences Drive, Davis, CA 95616, USA; msfiligenzi@ucdavis.edu (M.F.); rhpoppenga@ucdavis.edu (R.H.P.); 4 Pet Emergency and Specialty Center of Marin, 901 E. Francisco Blvd, San Rafael, CA 94901, USA; jenkaae@gmail.com

**Keywords:** lateral flow immunoassay, amatoxins, amanitins, point-of-care, mushroom poisoning

## Abstract

Globally, mushroom poisonings cause about 100 human deaths each year, with thousands of people requiring medical assistance. Dogs are also susceptible to mushroom poisonings and require medical assistance. Cyclopeptides, and more specifically amanitins (or amatoxins, here), are the mushroom poison that causes the majority of these deaths. Current methods (predominantly chromatographic, as well as antibody-based) of detecting amatoxins are time-consuming and require expensive equipment. In this work, we demonstrate the utility of the lateral flow immunoassay (LFIA) for the rapid detection of amatoxins in urine samples. The LFIA detects as little as 10 ng/mL of α-amanitin (α-AMA) or γ-AMA, and 100 ng/mL of β-AMA in urine matrices. To demonstrate application of this LFIA for urine analysis, this study examined fortified human urine samples and urine collected from exposed dogs. Urine is sampled directly without the need for any pretreatment, detection from urine is completed in 10 min, and the results are read by eye, without the need for specialized equipment. Analysis of both fortified human urine samples and urine samples collected from intoxicated dogs using the LFIA correlated well with liquid chromatography–mass spectrometry (LC-MS) methods.

## 1. Introduction

**Figure 1:**
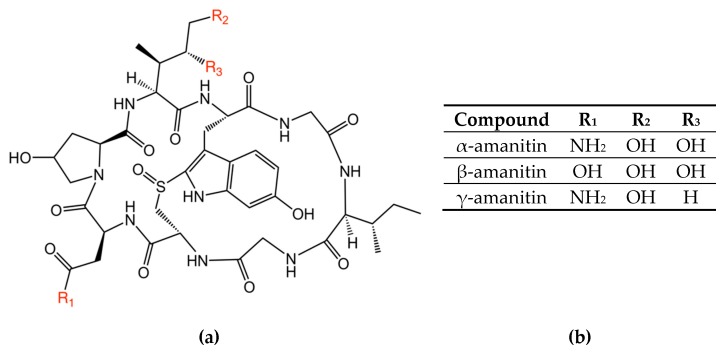
Chemical structures of the amatoxin variants examined in this paper, (**a**) molecular structure of the amanitin, (**b**) R-group designations for each variant.

Distinguishing toxic mushrooms from non-toxic ones is highly challenging, even for expert mycologists. Techniques to properly identify a mushroom include detailed morphological examination of the mushroom body, substrate identification, and knowledge of the location and the season. The toxins often associated with lethal cases are cyclopeptides, and more specifically amanitins (most commonly α-amanitin (α-AMA), β-AMA, and γ-AMA, collectively referred to as amatoxins) [1] (Figure 1). Amatoxins are found in a few species of mushrooms from different genera, including *Amanita*, *Galerina*, and *Lepiota* [2]. Amatoxins are highly resistant to degradation, and on the cellular level they inhibit transcription by binding to RNA polymerase II. As little as 0.1 mg/kg body weight of amatoxins may cause death [3,4], and this amount can be found in a single *Amanita phalloides*.

Consumption of toxin-containing mushrooms can result in a range of symptoms, from mild to life-threatening [5,6]. The presumptive diagnosis for amatoxin poisoning is based on a history of consuming wild mushrooms (if known), presentation of delayed gastroenteritis, elevated liver enzyme levels, and ruling out other gastrointestinal diseases or conditions [6]. To distinguish amatoxin poisonings, the presence of an amatoxin in an intoxicated patient’s urine would provide a definitive diagnosis. For dogs, obtaining a history of mushroom ingestion is rare, making diagnosis even more challenging. There are only a few laboratories capable of testing biological specimens for amatoxins to confirm human or animal exposures, and even when available, test results might not be available soon enough to help guide treatment. Although there are no FDA-approved antidotes, early diagnosis, aggressive immediate supportive care, and a range of potential therapies can potentially improve patient outcomes [6,7,8,9,10,11].

For both humans and dogs, the first symptoms of amatoxin poisonings usually appear 6–24 h after ingestion of an amatoxin-containing mushroom [6]. By this time, amatoxins have already begun damaging the liver and kidneys. Based on toxicological studies, amatoxins disappear rapidly from the serum, but are detectable in urine up to 4 days after ingestion [12,13,14]. In human urine, toxin concentrations decreased over time, and the highest concentrations observed were 4820 ng/mL for α-AMA and 7103 ng/mL for β-AMA [12]. Because of the relative ease of obtaining a urine sample, and the longer duration of detectability of amatoxins in urine compared to serum, urine seems an obvious sample matrix for performing rapid amatoxin analysis. Sensitive, rapid, and easy-to-perform methods are needed to detect amatoxins for the early diagnosis of toxin poisoning [9,15,16].

Current methods of chemical detection of amatoxins in urine include liquid chromatography–mass spectrometry (LC-MS) methods [17,18,19,20,21,22,23] and antibody-based enzyme-linked immunosorbent assays (ELISAs) [24,25]. LC-MS methods require sample extraction and expensive equipment, while ELISA methods require specialized equipment. Methods of both types typically take a few hours to complete. Lateral flow immunoassay (LFIA) formats utilize some of the reagents used for ELISA, but the entire test can be completed in minutes and requires no specialized equipment.

We have recently developed an LFIA for the detection of mushroom amatoxins [26]. As the sensitivity of the LFIA allows it to detect as little as 10 ng/mL, we hypothesized that this test would be useful for urine analysis in instances of mushroom poisonings. To test this hypothesis, we first conducted analysis of urine samples that were fortified with toxins (blind to the analyst) based on the reported concentrations of amatoxins identified in exposed individuals; and second, we used the LFIA to detect toxins in the urine samples collected from poisoned dogs. The LFIA results were compared to the established LC-MS methods [17,20]. Based on these studies, we can begin identifying the diagnostic utility of the LFIA for identifying amatoxin exposure.

## 2. Results and Discussion

### 2.1. Analytical Sensitivity of the LFIA and Interpretation of Results

**Figure 2:**
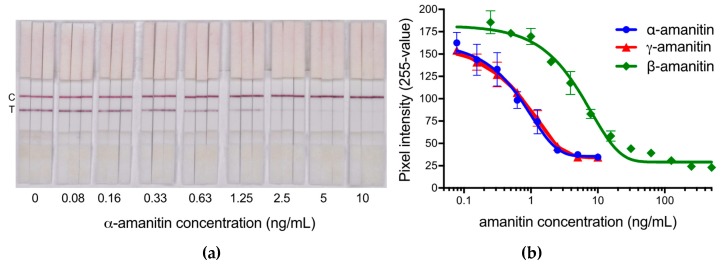
Standard curves for the detection of amatoxins by the lateral flow immunoassay (LFIA) in a pooled urine matrix. (**a**) A representative visual image of the LFIA test strips used for detecting α-amanitin (α-AMA). (**b**) Digitized values for the test line intensity for the detection of α-AMA, β-AMA, and γ-AMA. Data points represent the average of three replicates with error bars. T: test line, C: control line.

Standard curves were obtained for the detection of α-AMA, β-AMA, and γ-AMA in a pooled urine matrix (Figure 2). As this is a competitive binding assay, the test line signal intensity decreases with the increase in toxin concentrations. The cut-off value for each individual amanitin was determined by selecting the concentration where the test line almost completely disappears, which is equivalent to a pixel intensity value of approximately 30. For α-AMA and γ-AMA, the cut-off value is 10 ng/mL, while for β-AMA, the cut-off is 100 ng/mL. Although difficult to discern by eye, the limit of detection (defined as three times the standard deviation of a sample without amatoxins) is 0.3 ng/mL for α-AMA and γ-AMA and 1 ng/mL for β-AMA. To ensure consistent interpretation of the line intensity by eye, the cut-off values determined in this study were used for the remainder of this study to determine the diagnostic accuracy of the test for urine analysis.

**Figure 3:**
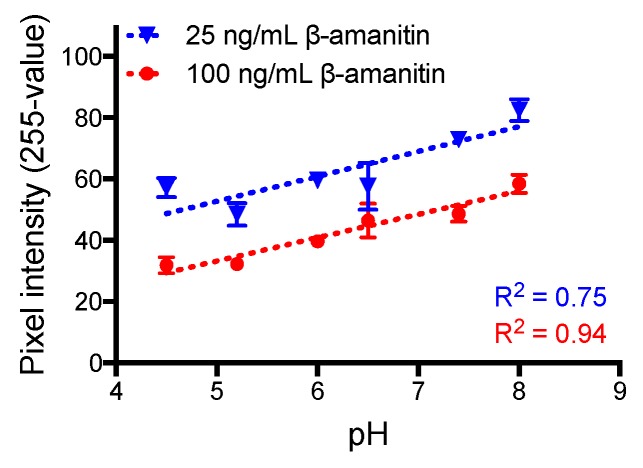
Test line intensities of the LFIA for solutions of β-amanitin in phosphate-buffered saline at different pHs.

The cut-off value for β-AMA when detected in urine resulted in a 20-fold more sensitive assay cut-off value than our previous standard curve developed in phosphate-buffered saline (PBS) [26]. There was no change in sensitivity for α-AMA or γ-AMA when using urine or PBS as the matrix [26]. To test whether the β-AMA sensitivity differences were possibly due to the pH of the matrix, we evaluated pH-adjusted PBS buffers ranging from 4.5 to 8 (Figure 3). Indeed, there was a positive trend for the two concentrations of β-AMA tested (100 and 25 ng/mL), in which the test line intensity increased with the increase in pH. This trend corroborated with the differences between the pH of urine and that of PBS. The pH of the pooled urine matrix was 6.0, while the pH of PBS was 7.4.

### 2.2. Detection of Amatoxins in Blind Fortified Human Urine Samples

**Figure 4:**
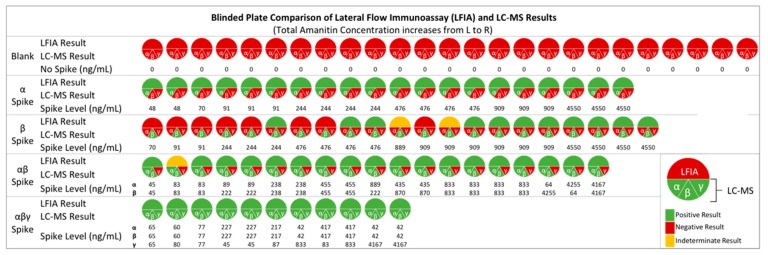
Design and results of experiments on amatoxin-fortified human urine samples. The toxin concentrations shown are the nominal spiked amounts. LC-MS: liquid chromatography–mass spectrometry, L: left, R: right.

We recapitulated a human exposure study by spiking unexposed human urine samples with concentrations (45 to 4550 ng/mL) of amatoxins measured from actual food exposures [12]. Two modifications were made to the sample set: (1) we included γ-AMA along with α-AMA and β-AMA, and (2) we utilized a mixture of single and pooled urine samples. LFIA analysis was conducted as a blind test so that the LFIA readers would utilize only the LFIA as the detection method to see how well they could identify amatoxin-containing urine samples. All samples were also validated using an LC-MS/MS method [17] for confirmation. Figure 4 shows the nominal spiked concentrations along with LFIA and LC-MS/MS results.

In this sample set, the diagnostic efficiency of the LFIA for indicating when a sample contained amatoxins and when it did not was outstanding (94.6%) (Table 1). Diagnostic sensitivity (true positive rate) was 92.3% and specificity (true negative rate) was 100%. These diagnostic descriptor calculations took into account the cut-off values determined for each analyte (i.e., 10 ng/mL for α-AMA and γ-AMA, and 100 ng/mL for β-AMA). Overall, there were only three samples that were recorded as indeterminate due to the difference in interpretation by two independent readers.

Using the cut-off values for each analyte compared to the nominal spike value resulted in a designation of “false negative” for only five samples. A false negative result meant that the LFIA reported a negative result, although the nominal concentration was greater than the cut-off value that should have resulted in a positive detection result. All five false negative designations occurred in samples that contained only β-AMA in the amount of 244–909 ng/mL (Figure 4). Of these five incorrectly identified samples, three were from single urine samples, while the other two were from the pooled urine matrix. This error is plausibly due to the interpretation of a faint test line as the toxin concentration approaches the cut-off value of the assay.

Interestingly, 7 samples fortified with only β-AMA in the range of 244–909 ng/mL tested positive and were reported as true positive, since they matched the criteria of being above the assay’s cut-off value. All the other samples fortified with only β-AMA below this range (i.e., 91 ng/mL or lower; *n* = 3) tested negative, and the samples that were above this range (i.e., 4550 ng/mL; *n* = 4) tested positive, as expected. Overall, inaccuracy of LFIA results is observed when only β-AMA is detected at a level close to its cut-off value. For α-AMA and γ-AMA, the concentrations tested were all at least 4-fold higher than the cut-off value, since these concentrations were those that were previously reported for amatoxin poisoning cases, and thus are clinically relevant. Detecting low concentrations of β-AMA may appear to be a limitation of this technology; however, α-AMA and β-AMA are almost always found at comparable concentrations in mushrooms [27,28], and thus either analyte serves as a good biomarker for determining amatoxin poisoning.

Most previous amatoxin exposure studies measured α-AMA and sometimes β-AMA, however γ-AMA was often not included. Although the kinetics of γ-AMA are not well studied, we sought to include it because it could conceivably be a diagnostic marker of amatoxin poisoning. The previously described studies relying on immunoanalytical methods might also have been detecting γ-AMA, because their reagents cross-reacted with this analyte [25]. Furthermore, the previously described immunoanalytical methods were less sensitive to β-AMA [29], thus those tests would have also missed the samples that this LFIA missed when only β-AMA was present in a urine sample.

The use of pooled and single urine samples was meant as a means to identify any potentially interfering components found in urine. In the instances of inaccuracy mentioned above, no differences were attributed to those samples being a single or a pooled urine sample. Furthermore, there were no false positives observed in this amatoxin-fortified human urine study, which means that when a sample had no amatoxins below the defined threshold concentration, it was interpreted correctly by the LFIA. Together, these results underscore that no apparent urine components (natural or potentially synthetic—urine samples were neither subjected to drug screening nor were they deemed free of drugs) interfere with amatoxin detectability.

### 2.3. Detection of Amatoxins in Dog Urine Samples

Unfortunately, many dogs each year are poisoned by amatoxins due to their natural curiosity and indiscriminate eating habits [30,31]. To evaluate this LFIA for diagnostic potential, we collaboratively analyzed samples submitted to the California Animal Health and Food Safety Laboratory in Davis, CA, USA (CAHFS Davis). Urine samples were qualitatively analyzed both by LFIA and LC-MS/MS/MS methods [20] (Table 1). These samples were collected from the dogs presumed to have ingested amatoxin-containing mushrooms and from the dogs not suspected of mushroom poisoning (a mix of healthy and sick dogs).

The LC-MS/MS/MS method only detects α-AMA. The LC-MS/MS/MS result reports “positive” if the sample has detectable (above 1 ng/mL) amounts of α-AMA, reports “negative” when no amounts are detected, and reports “trace” when a feature is detected with the correct retention time, molecular weight, and fragmentation pattern, but the concentration is below 1 ng/mL.

**Figure 5:**
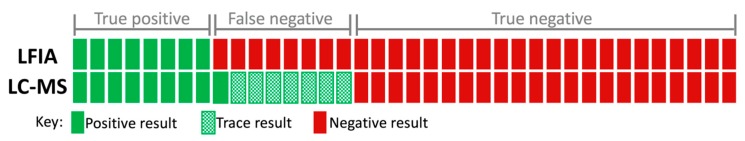
Comparison of methods (LC-MS/MS/MS and LFIA) for determining the presence of amatoxins in intoxicated dog urine samples (*n* = 38).

As shown in Figure 5, the LFIA should and does indicate negative when the LC-MS/MS/MS indicates trace (*n* = 7), since the limit of detection for the LC-MS/MS/MS is 1 ng/mL, which is 10-fold lower than for the LFIA. Only one sample was found to be negative by the LFIA and positive by the LC-MS/MS/MS. This particular sample had an estimated LC-MS/MS/MS concentration of 2 ng/mL, which is below the LFIA’s cut-off limit (10 ng/mL), but above the threshold for positive for the LC-MS/MS/MS (1 ng/mL). However, for the rest of the samples that had either detectable amounts of α-AMA as determined by the LC-MS/MS/MS (i.e., true positive, *n* = 8) or non-detectable amounts of α-AMA as determined by the LC-MS/MS/MS (i.e., true negative, *n* = 22), the LFIA correlates 100% (Table 1). Based on this consistency, there seems to be no components in dog urine that interfere with generating reliable results. Based on this dog urine sample set, the calculated values for diagnostic sensitivity (50%), specificity (100%), and efficiency (78.9%) are provided in Table 1. This sample set distribution is not the representative distribution of prevalence of the poisonings encountered in the population, and so a larger sample size would help to determine diagnostic characteristics more accurately.

All detection methods have their benefits and limitations. LC-MS methods can provide a more definitive analysis for the samples containing lower concentrations of amatoxins, but all samples (e.g., urine and mushrooms) require sample extraction before detection. This LFIA method is exceptionally rapid (10 min), requires no sample extraction for urine, and the test is portable. In the mushroom poisoning scenarios where the illness progresses rapidly, the LFIA described here is distinctively appropriate for point-of-care urine testing. The speed of analysis and lack of requirement for trained personnel and expensive instrumentation make this an ideal point-of-care method. Because there is no clinical tool to determine amatoxin poisoning, this LFIA test should be further exploited given its reliable diagnostic performance in this study.

## 3. Materials and Methods

### 3.1. Materials and Approvals

All the unexposed human urine samples were obtained from a commercial provider (Tennessee Blood Services, Memphis, TN, USA) and pre-screened by the vendor in accordance with FDA regulations, and thus no consent procedures were required for this project (IRB #201210385). All animal urine samples were collected and submitted by the owners with their consent or by their veterinarians to the California Animal Health and Food Safety Laboratory System, Davis, and thus did not require the Institutional Animal Care and Use Committee review.

**Figure 6:**
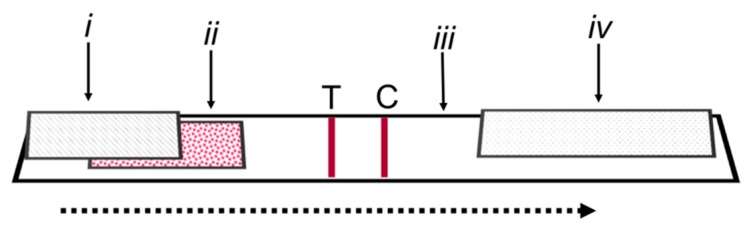
Schematic diagram of the lateral flow strip components: (i) sample pad, (ii) conjugate pad, (iii) nitrocellulose membrane, (iv) wicking pad, (T) test line, (C) control line. The arrow indicates the flow direction.

Monoclonal antibody (AMA9G3), hapten-protein conjugate (LB-AMA-BSA), and full LFIA test strips were produced as described earlier [26,32,33]. The LFIA components are diagramed in Figure 6. The standards used were α-amanitin (α-AMA; ≥90%, Enzo Life Sciences, Farmingdale, NY, USA), β-AMA (≥90%, Enzo), and γ-AMA (≥90%, Enzo).

### 3.2. Analytical Sensitivity of the LFIA

The sensitivity of the LFIA for α-AMA, β-AMA, and γ-AMA was determined using a pooled urine matrix. The urine matrix for calibration curves was generated using 10 single urine samples pooled together. All 10 single urine samples were tested separately as negative controls and confirmed to not interfere with the read-out. Two-fold dilutions of the standards ranging from 0.08 to 2000 ng/mL were made using the pooled urine as the diluent. Then, 100 µL of each concentration were applied to the sample pad region of the test strip and tested in triplicate. The signal intensity of both the control and test lines was resolved in 10 min. If no visible control line appeared on the test strip, the test was determined to be invalid. Digital photographs of the test strips were obtained using a Nikon SLR camera equipped with an LED ring light (B&H Foto and Electronics Corps, New York, NY, USA) for even lighting. The images were analyzed using ImageJ software (NIH, Bethesda, MD, USA). Images were contrast-enhanced (default setting of 0.3%), and boxes of consistent size were used to integrate the test line’s pixel value. Pixel values were inverted by subtracting the measured value from the maximum possible (i.e., 255). Data was plotted using GraphPad Prism 7 software (GraphPad Software, San Diego, CA, USA) using a four-parameter logistic equation.

PBS solutions (10 mM phosphate, 138 mM NaCl, 2.7 mM KCl, pH 7.4) were adjusted to pH 4.5, 5.2, 6, 6.5, 7.4, and 8 by adding either 1 M NaOH or 1 M HCl. Each solution was then spiked with β-AMA at 100 and 25 ng/mL. As described above, 100 µL of each solution were applied to the sample pad region of the test strip, tested in triplicate, incubated for 10 min, photographed, and interpreted by digital analysis.

### 3.3. Amatoxin-Fortified Human Urine Analysis

Single (*n* = 48) and pooled (*n* = 48) human urine samples (obtained under IRB #201210385) were fortified (blind to the LFIA researchers) with varying amounts of α-AMA, β-AMA, and γ-AMA. A 96-well microtiter plate was prepared by spiking urine with α-AMA, β-AMA, and γ-AMA at concentrations ranging from 45 to 4550 ng/mL following earlier findings from exposed individuals [12]. The distribution of samples was intended to follow these findings such that 19 wells contained α-AMA and β-AMA, 20 wells contained only α-AMA, 21 wells contained only β-AMA, 11 wells contained α-AMA, β-AMA, and γ-AMA, and 25 wells contained no amatoxins. These spiked concentrations were randomly distributed between the single and pooled urine matrices.

For analytical confirmation, all human urine samples were also analyzed following the previously validated LC-MS/MS method used for human urine [17]. The LFIA method was performed by placing 100 µL of a urine sample directly onto the sample pad and waiting for approximately 10 min before interpreting the line intensity of the test line. A visual qualitative reading of either YES(+) or NO(–) (YES – no visible test line, NO – a visible test line) was performed by two individuals, and a digital image of the strip was also acquired. If no control line appeared, then the test was determined to be invalid. The LFIA’s cut-off values were set at 10 ng/mL for α-AMA and γ-AMA, and at 100 ng/mL for β-AMA. If amatoxin concentrations exceeded these values, the result was to read positive (defined as true positive), and if the amatoxin concentrations were below these cut-off values, the result was to read negative (defined as true negative). On the basis of these studies, diagnostic sensitivity (true positive rate), diagnostic specificity (true negative rate), and diagnostic efficiency of the LFIA results were calculated using the following formulae:
False positive (FP) =LFIA positive, although the amatoxin concentration was below the cut-off value False negative (FN) =LFIA negative, although the amatoxin concentration was above the cut-off valueDiagnostic sensitivity =TP/(TP + FN)Diagnostic specificity =TN/(TN+ FP)Single-study diagnostic efficiency =(TP + TN)/(TP

### 3.4. Collected Dog Urine Analysis for Amatoxins

Urine samples of the dogs with suspected amatoxin intoxication were submitted by their owners or by their veterinarians. For analytical confirmation, dog urine samples were analyzed following the previously validated LC-MS/MS/MS method used for dog urine [20]. The LFIA method was performed as described previously for human urine samples by placing 100 µL of a urine sample directly onto the sample pad and waiting for approximately 10 min before interpreting the line intensity of the test line.

## Figures and Tables

**Table 1:** Performance of LFIA for qualitative determination of the presence of amatoxins in blind fortified urine samples and collected dog urine samples.

Diagnostic Parameter	Fortified Human Urine ^a^	Intoxicated Dog Urine ^b^
# of samples	*n* = 96 ^c^	*n* = 38
True positive (TP)	60	8
True negative (TN)	28	22
False positive (FP)	0	0
False negative (FN)	5	8
Sensitivity	92.3%	50%
Specificity	100%	100%
Efficiency	94.6%	78.9%

^a^ Compared to the LC-MS/MS method [17] for confirmation. ^b^ Compared to LC-MS/MS/MS method [20] for confirmation. ^c^ 3 samples were not included in this analysis, because the LFIA results obtained by two independent readers were ambiguous.
